# The global impact of industrialisation and climate change on antimicrobial resistance: assessing the role of Eco-AMR Zones

**DOI:** 10.1007/s10661-025-14086-3

**Published:** 2025-05-05

**Authors:** Emmanuel Adedeji Oyelayo, Tayo John Taiwo, Samuel Oyeponle Oyelude, Jude Oluwapelumi Alao

**Affiliations:** 1 Department of Biochemistry, Ladoke Akintola University of Technology, Ogbomosho, Nigeria; 2 Department of Medicine, Ladoke Akintola University of Technology, Ogbomosho, Nigeria; 3 School of Public Health and Interdisciplinary Studies, Auckland University of Technology, Auckland, New Zealand

**Keywords:** Antimicrobial resistance (AMR), Climate change, Industrialisation, Eco-AMR Zones, Environmental sustainability

## Abstract

This study examines the relationship between industrialisation, climate change, and antimicrobial resistance (AMR) gene prevalence. Data analysis from the top 20 highly industrialised and the top 20 least industrialised nations revealed that industrial activities significantly contribute to global warming, with temperature increases of up to 2 °C observed in highly industrialised regions. These environmental changes influence the distribution and evolution of AMR genes, as rising temperatures can affect bacterial resistance in a manner similar to antibiotics. Through a bioinformatics approach, a marked disparity in AMR gene frequencies was observed between highly industrialised and less industrialised nations, with developed countries reporting higher frequencies due to extensive antibiotic use and advanced monitoring systems. ‘Eco-AMR Zones’ is proposed as a solution to specialised areas by promoting sustainable industrial practices, enforcing pollution controls, and regulating antibiotic use to mitigate AMR’s environmental and public health impacts. These zones, supported by collaboration across various sectors, offer a promising approach to preserving antibiotic effectiveness and reducing environmental degradation. The study emphasises the importance of integrated global strategies that address both the ecological and public health challenges posed by AMR, advocating for sustainable practices, international collaboration, and ongoing research to combat the evolving threats of climate change and antimicrobial resistance.

## Introduction

Antimicrobial resistance (AMR) arises when microorganisms such as bacteria, fungi, viruses, and parasites develop mechanisms to withstand antimicrobial agents. Although AMR occurs naturally in some cases, human activities—particularly the overuse and misuse of antimicrobials in human and veterinary medicine—have significantly accelerated its emergence and spread (Gahamanyi et al., 2021; Kimera et al., 2020). The escalating burden of AMR poses a severe global threat. Projections indicate that by 2050, AMR could account for 10 million deaths annually and impose economic costs of up to USD 100 trillion without effective interventions (Burki, 2018). In 2019 alone, AMR directly caused an estimated 1.3 million deaths, exceeding the fatalities from HIV/AIDS, breast cancer, and malaria combined (Murray et al., 2022).

Beyond antimicrobial misuse, environmental factors such as climate change and industrialisation play a crucial role in shaping the dynamics of AMR. Recent studies highlight the intricate, bidirectional relationship between climate change and AMR. For instance, Saffiatou Darboe et al. (2020) identified human activities—particularly excessive antibiotic use—as a key global driver of AMR. Climate change exacerbates this issue by altering environmental conditions that facilitate the persistence and dissemination of resistant bacteria. Rising global temperatures, changing precipitation patterns, and extreme weather events—according to Ebi et al. (2021)—disrupt sanitation systems, promote pathogen transmission, and create conditions conducive to resistance development. Similarly, Van Eldijk et al. (2024) demonstrated that warming temperatures in agricultural settings accelerate bacterial resistance due to increased antibiotic use and enhanced mutation rates.

Industrialisation further compounds these challenges by contributing to environmental pollution and greenhouse gas emissions, exacerbating the AMR impacts driven by climate change. Industrial activities release unregulated by-products, including pharmaceutical residues and heavy metals, contaminating both aquatic and terrestrial ecosystems. These contaminants create selective pressures that drive bacterial adaptation and resistance, particularly in environments such as freshwater bodies and soil ecosystems (Li et al., 2024). In conjunction with climate change, the ecological disruptions caused by these activities underscore the complexity of AMR mitigation (Magnano San Lio et al., 2023).

Critical research gaps persist despite growing recognition of the links between AMR, climate change, and industrialisation. While previous studies have examined these factors in isolation, a comprehensive framework that integrates these dimensions to inform effective mitigation strategies remains lacking. The current literature also fails to propose solutions that simultaneously address environmental sustainability and AMR containment. This study seeks to bridge these gaps by exploring integrated strategies that align climate adaptation with AMR mitigation, providing a dual-benefit approach for public health and environmental management.

Additionally, regional disparities in AMR drivers and impacts remain underexplored. While industrial by-products and climate change contribute to AMR globally, their effects vary across regions with differing levels of industrialisation. This study will compare highly industrialised and less industrialised areas to assess their relative contributions to AMR prevalence and the distribution of resistance genes. Using *Escherichia coli* as a model organism, this research will analyse industrial activities’ environmental and health impacts, identifying global trends associated with varying degrees of industrialisation.

By addressing these gaps, this study aims to advance the understanding of the complex interconnections between AMR, climate change, and industrialisation. The findings will contribute to developing innovative, regionally tailored strategies to mitigate AMR within the broader context of global environmental challenges.

## Methods

### Data collection and industrial landscape analysis

A multi-source data approach was utilised to assess the industrial landscape and environmental impact of the top 20 highly industrialised and top 20 poorly industrialised countries in 2023. The identification of the top manufacturing countries was based on industrial activity and output data sourced from the World Bank and compiled by Yahoo! Finance (Haqqi, 2023). The ranking of countries was determined by factors such as GDP contribution from manufacturing, the number of key manufacturing sectors, and the overall scale of industrial production.

To analyse the impact of climate change in these countries, we referred to the ‘Berkeley Earth’s 2023 Global Temperature Report’, a publication by Berkeley Earth, a California-based non-profit organisation specialising in land temperature data analysis for climate science (Rohde, 2024). This report was instrumental in examining the extent of global warming experienced by these nations in 2023 relative to their average temperatures from 1951 to 1980. Additionally, temperature changes across different continents due to climate change were compared using data from the Food and Agriculture Organization Corporate Statistical Database (FAOSTAT) (FAOSTAT, 2024), which provided temperature data for specific regions, allowing for the comparison of climate change effects in countries with varying levels of industrialisation.

### Prevalence assessment of AMR genotypes

To investigate the prevalence of AMR genotypes in *E. coli* across the selected countries, genetic data was sourced from the National Centre for Biotechnology Information (NCBI). This source was chosen due to its comprehensive repository of microbial genomic information, which enabled us to identify patterns of AMR in both highly industrialised and less industrialised nations.

For comparative purposes, data on less industrialised African countries were also analysed using information from the African Development Bank (2022). This comparison allowed contextualisation of the prevalence of AMR genotypes in *E. coli* within the framework of varying levels of industrialisation.

### Bioinformatics approach for AMR genotype analysis

The bioinformatics analysis was conducted through the NCBI pathogen detection portal, following a systematic procedure to ensure comprehensive data extraction and accuracy. We selected *E. coli* as the focus organism due to its well-documented resistance patterns and availability of extensive genetic data. Advanced filtering tools on the NCBI platform were used to refine the search by ‘location’ and ‘AMR genotypes’, ensuring that the data collected was highly relevant to the study’s aims. The dataset was extracted from NCBI between 1905 and 2023, providing a comprehensive temporal perspective on AMR gene distribution. Filters for location and specific AMR genotypes were applied to further enhance the relevance and precision of the data for the top 20 highly industrialised and top 20 poorly industrialised countries, allowing for a direct comparison of AMR genotype prevalence.

The specific ‘AMR genotypes’ identified in this study describe the genetic configurations that include resistance genes, which are crucial for understanding the bacterial resistance to various antimicrobial agents.

Quantitative data analysis focused on the total number of genes screened, the proportion identified as AMR genes, and their classification into categories such as:

1. Complete AMR genes (CAGs): Fully functional genes that allow bacteria to resist specific antibiotics completely.
2. Point AMR genes (PAGs): Genes with specific point mutations in the bacterial DNA that confer antibiotic resistance.
3. Partial end contig AMR genes (PECAGs): Incomplete genetic sequences found at the ends of DNA contigs, associated with antibiotic resistance.
4. Partial AMR genes (PARGs): Incomplete or fragmented genes that still contribute to some level of antibiotic resistance.
5. Mistranslation AMR genes (MAGs): Genes that result from errors during protein synthesis, producing altered proteins that can impact bacterial responses to antibiotics.

### Correlation analysis

A correlation matrix was generated to evaluate the relationships between different types of AMR genes, specifically focusing on CAGs, PAGs, PARGs, MAGs, and PECAGs. This analysis was conducted to identify which gene types are most closely associated with CAGs, and to determine the strength of these associations. The purpose was to establish a foundational understanding of how these variables are interrelated before proceeding to more complex modelling.

### Regression analysis

A linear regression model was employed to quantify the impact of each AMR gene type on CAGs. The goal was to determine each independent variable’s predictive power and confirm the findings from the correlation analysis. The regression coefficients provided insight into how each AMR gene type changes could influence the number of CAGs. This method was chosen to assess the relative importance of each gene type in a predictive framework.

### Model performance and diagnostics

The performance of the regression model was evaluated using the intercept, R-squared value, and diagnostic plots. The intercept indicated the baseline level of CAGs, while the R-squared value assessed how well the model explained the variance in CAGs. Q-Q plots were used to check the assumptions of the regression model, particularly the normality of residuals, ensuring the robustness and validity of the model.

### Random forest model

The Random Forest model was used alongside regression analysis to enhance the study’s ability to analyse AMR-related data. Unlike regression, Random Forest handles non-linear relationships and interactions, providing a deeper understanding of the factors influencing AMR gene prevalence. It also identifies key predictors through feature importance analysis and validates the regression findings using performance metrics like R-squared and MSE. This dual-method approach ensures robust, comprehensive insights into the drivers of AMR.

## Results

### Comparative analysis of industrialisation levels across selected nations

The findings reveal stark disparities in industrial activity by comparing the manufacturing output rankings of the top 20 highly industrialised countries (T20HINW) and the top 20 poorly industrialised nations in Africa (T20PINA). Table 1 highlights these differences, establishing a foundational context for analysing AMR gene prevalence and environmental impacts.

**Table 1:** Comparative rankings of the top 20 global manufacturing nations and the 20 least industrialised nations in Africa

Ranking	T20HINW	T20PINA
1	China	Nigeria
2	United States of America	Ghana
3	Germany	Côte d'Ivoire
4	South Korea	Senegal
5	India	Kenya
6	Italy	Tanzania
7	United Kingdom	Uganda
8	France	Ethiopia
9	Russia	Djibouti
10	Mexico	Rwanda
11	Indonesia	Burkina Faso
12	Turkey	Benin
13	Ireland	Guinea
14	Spain	Togo
15	Brazil	Liberia
16	Switzerland	Sierra Leone
17	Thailand	Comoros
18	Poland	Burundi
19	Netherlands	Eritrea
20	Saudi Arabia	Somalia

### Comparison of global warming impact in 2023: highly vs. poorly industrialised nations

The analysis of temperature increases in 2023 (Tables 2 and 3) provides insights into how industrialisation correlates with the intensity of climate change. Highly industrialised nations experienced greater mean temperature increases (0.8–2.6 °C) than poorly industrialised nations (1.2–1.8 °C), suggesting that regions contributing more to greenhouse gas emissions also face more severe global warming effects. This finding supports the hypothesis that industrialisation intensifies environmental challenges, including those that may exacerbate AMR dynamics.

**Table 2:** 2023 global warming impact on top 20 highly industrialised nations in the world

Ranking	T20HINW	2023 global warming experience for T20HINW (°C)
1	China	1.9
2	United States of America	1.4
3	Germany	2.4
4	South Korea	1.8
5	India	0.8
6	Italy	2.2
7	United Kingdom	1.7
8	France	2.4
9	Russia	2.3
10	Mexico	1.2
11	Indonesia	1.1
12	Turkey	1.8
13	Ireland	1.8
14	Spain	2.1
15	Brazil	1.5
16	Switzerland	2.6
17	Thailand	1.3
18	Poland	2.4
19	Netherlands	2.3
20	Saudi Arabia	1.7

**Table 3:** 2023 global warming impact on top 20 poorly industrialised nations in Africa (T20PINA)

Ranking	T20PINA	2023 global warming experience for T20PINA (°C)
1	Nigeria	1.4
2	Ghana	1.2
3	Côte d'Ivoire	1.3
4	Senegal	1.8
5	Kenya	1.4
6	Tanzania	1.4
7	Uganda	1.4
8	Ethiopia	1.3
9	Djibouti	1.2
10	Rwanda	1.2
11	Burkina Faso	1.4
12	Benin	1.3
13	Guinea	1.2
14	Togo	1.3
15	Liberia	1.3
16	Sierra Leone	1.3
17	Comoros	1.5
18	Burundi	1.4
19	Eritrea	1.3
20	Somalia	1.4

### Statistical analysis of global warming impact in selected nations

To further examine global warming trends, a statistical comparison revealed higher variability (SD = 0.49) among highly industrialised nations than poorly industrialised ones (SD = 0.13). This trend underscores the unequal distribution of climate change impacts, a critical factor for understanding regional differences in AMR prevalence.

### Regional temperature variations and global warming trends (2000–2022)

**Fig. 1:**
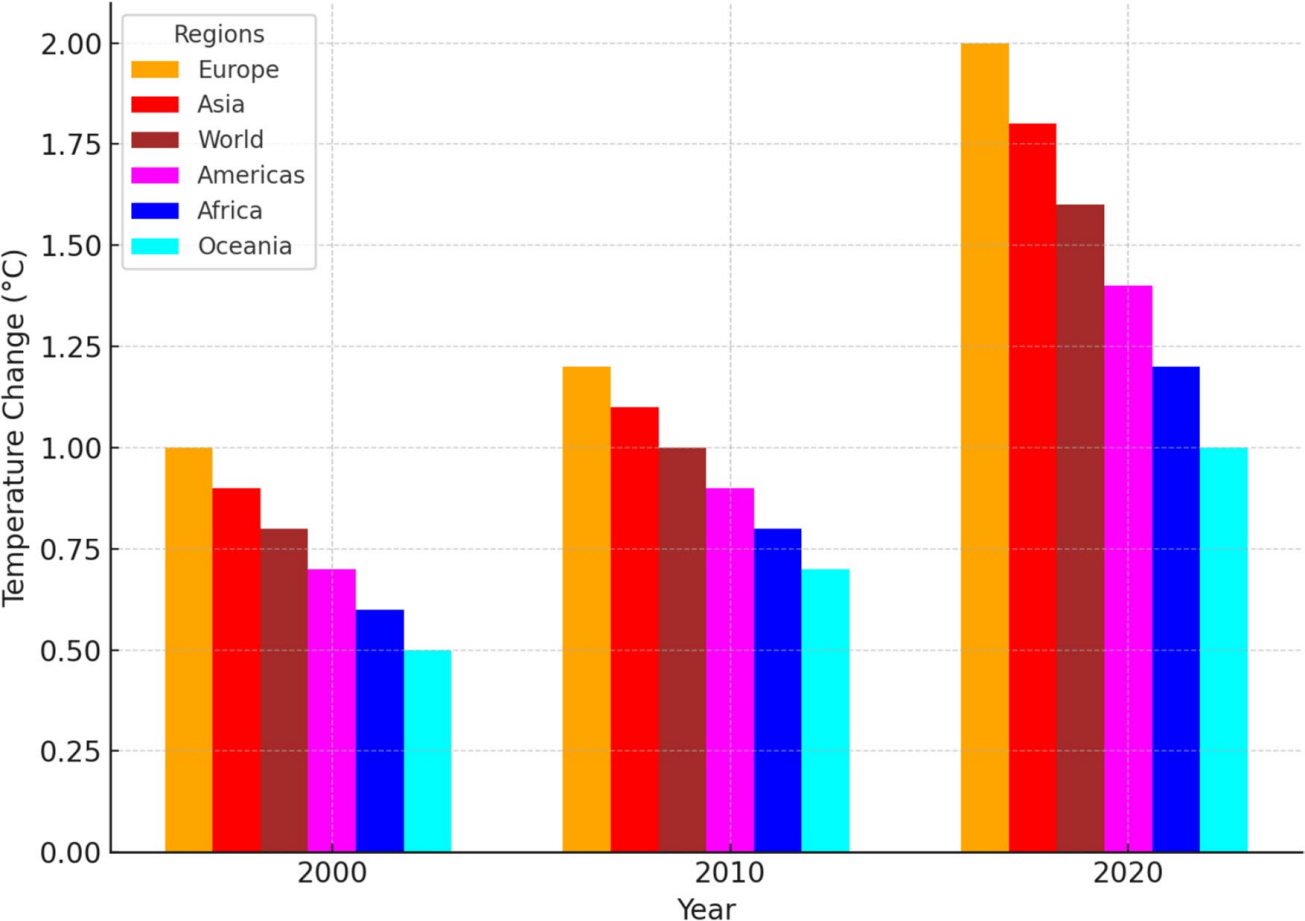
Regional variations in land temperature change over time

Figure 1 illustrates the regional trends in land temperature changes over two decades, highlighting Europe with the highest temperature increases. This aligns with the study’s objective of identifying areas most impacted by industrialisation-related environmental changes, setting the stage for linking these changes to AMR proliferation.

### Prevalence and diversity of AMR genes in nations under consideration

The prevalence and diversity of AMR genes (Tables 4 and 5) directly address the research question about the genetic dynamics of AMR across industrialised and non-industrialised regions. Highly industrialised nations exhibit a significantly higher prevalence of AMR genes, with the USA leading in all categories. Highly industrialised nations account for 570,375 AMR genes, while poorly industrialised nations contribute 13,155 AMR genes, bringing the overall dataset to 583,530 AMR genes. Conversely, poorly industrialised nations show a less pronounced but still concerning distribution of AMR genes. These findings suggest that industrialisation plays a critical role in AMR proliferation through direct pollution and indirect climate-related effects.

**Table 4:** Frequency of AMR genes in the top 20 highly industrialised nations in the world

Country	CAGs	PAGs	PECAGs	PARGs	MAGs
China	15,282	14,271	5108	1705	3490
USA	130,583	125,607	28,940	8928	11,991
Germany	5591	5122	1154	662	242
South Korea	917	847	136	136	163
India	1471	1376	247	212	146
Italy	900	847	150	92	85
United Kingdom	42,874	40,647	44,155	3585	14,225
France	7957	7481	1524	898	405
Russia	598	570	164	65	162
Mexico	362	316	36	44	23
Indonesia	72	57	14	7	5
Turkey	159	151	65	25	10
Ireland	925	852	138	78	158
Spain	2445	2,224	564	225	150
Brazil	895	831	225	121	64
Switzerland	2936	2,761	630	326	108
Thailand	1959	1764	863	274	95
Poland	505	454	143	70	41
Netherlands	6671	6252	1656	967	668
Saudi Arabia	157	142	46	27	9

**Table 5:** Frequency of AMR genes in top 20 poorly industrialised nations in Africa

Country	CAGs	PAGs	PECAGs	PARGs	MAGs
Nigeria	764	695	308	116	32
Ghana	260	242	68	35	5
Côte d'Ivoire	1	1	N/A	N/A	N/A
Senegal	16	10	2	1	1
Burkina Faso	56	54	33	8	9
Guinea	60	57	2	9	6
Benin	29	29	18	6	2
Togo	4	4	N/A	1	N/A
Sierra Leone	N/A	N/A	N/A	N/A	N/A
Liberia	1	N/A	N/A	N/A	N/A
Kenya	2346	1968	480	476	137
Tanzania	1096	936	150	178	15
Ethiopia	219	202	117	87	4
Uganda	553	522	182	72	N/A
Rwanda	150	144	104	33	7
Djibouti	1	1	1	N/A	N/A
Eritrea	N/A	N/A	N/A	N/A	N/A
Somalia	9	9	2	1	5
Burundi	1	1	1	N/A	N/A
Comoros	N/A	N/A	N/A	N/A	N/A

### Correlation analysis

**Fig. 2:**
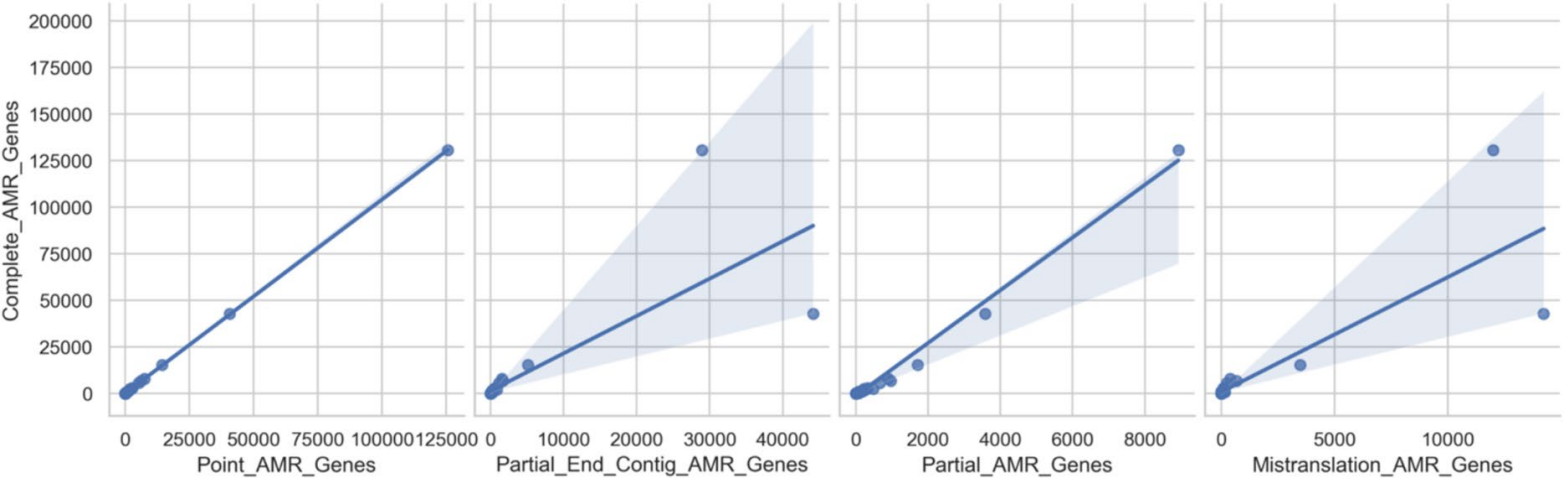
Correlation analysis of AMR gene types in predicting complete AMR genes

The correlation matrix (Fig. 2) identifies strong positive relationships between AMR gene types, with PAGs and CAGs showing near-perfect correlation (*r* = 0.999982). This supports the hypothesis that specific AMR gene types are primary predictors of overall AMR prevalence, forming a basis for targeted mitigation strategies.

### Regression analysis

**Fig. 3:**
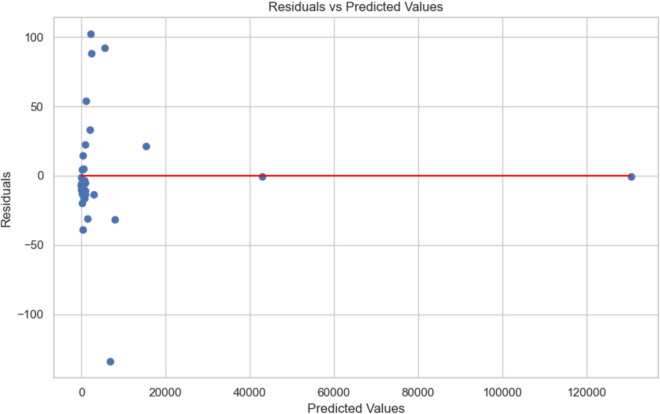
Diagnostic plots for correlation analysis of AMR gene predictions

The regression model (Fig. 3) reinforces the significance of PAGs and PARGs as key predictors of CAGs, with coefficients of 0.997 and 0.570, respectively. These findings validate the hypothesis that certain gene types disproportionately influence AMR dynamics.

### Model performance

**Fig. 4:**
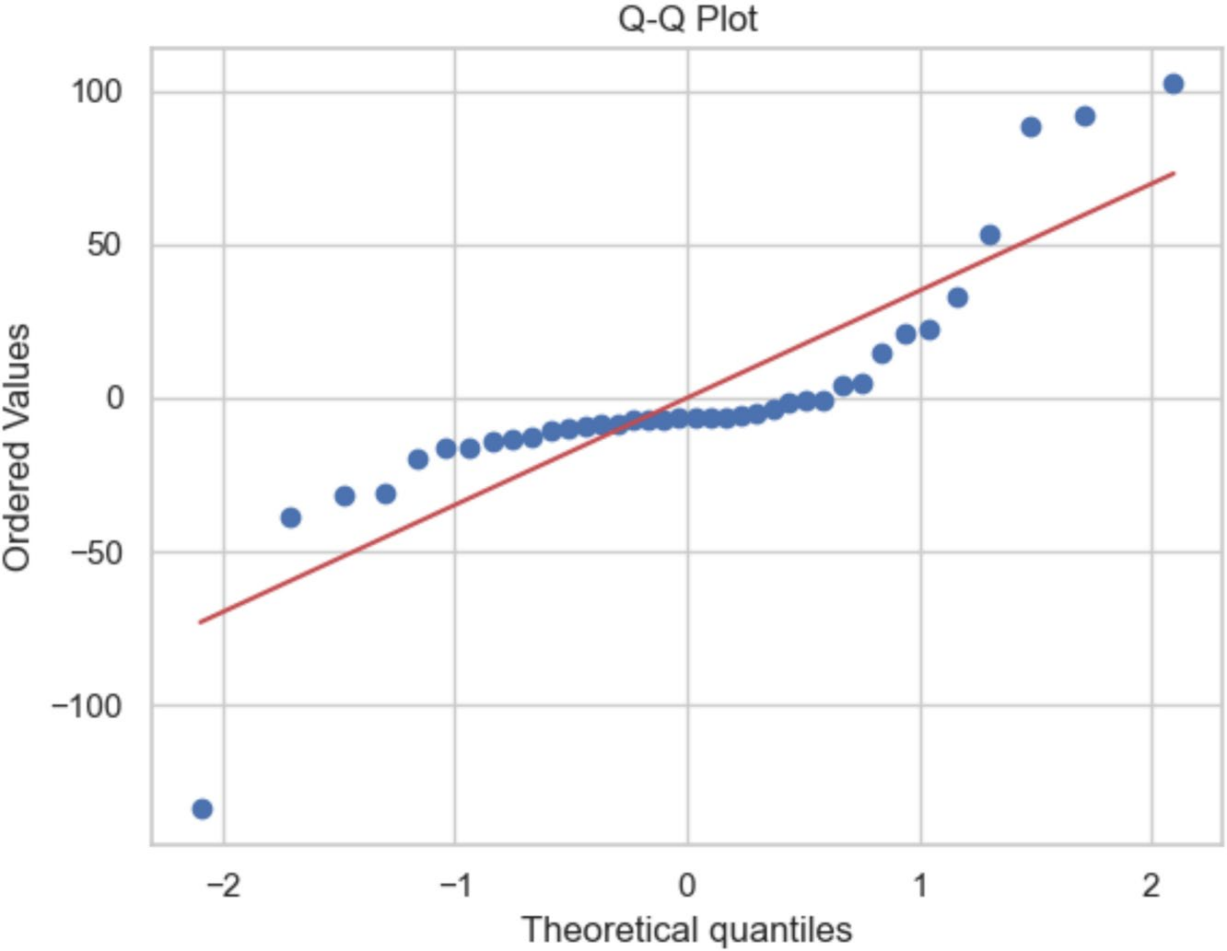
Q-Q plot for assessing normality of residuals in AMR gene prediction model

The regression model’s R-squared value (0.999997) and residual normality (Fig. 4) confirm its reliability in explaining AMR gene prevalence. This high explanatory power indicates that the independent variables selected effectively capture the key drivers of AMR dynamics, addressing the hypothesis on environmental and industrial predictors.

### Random forest model performance

**Fig. 5:**
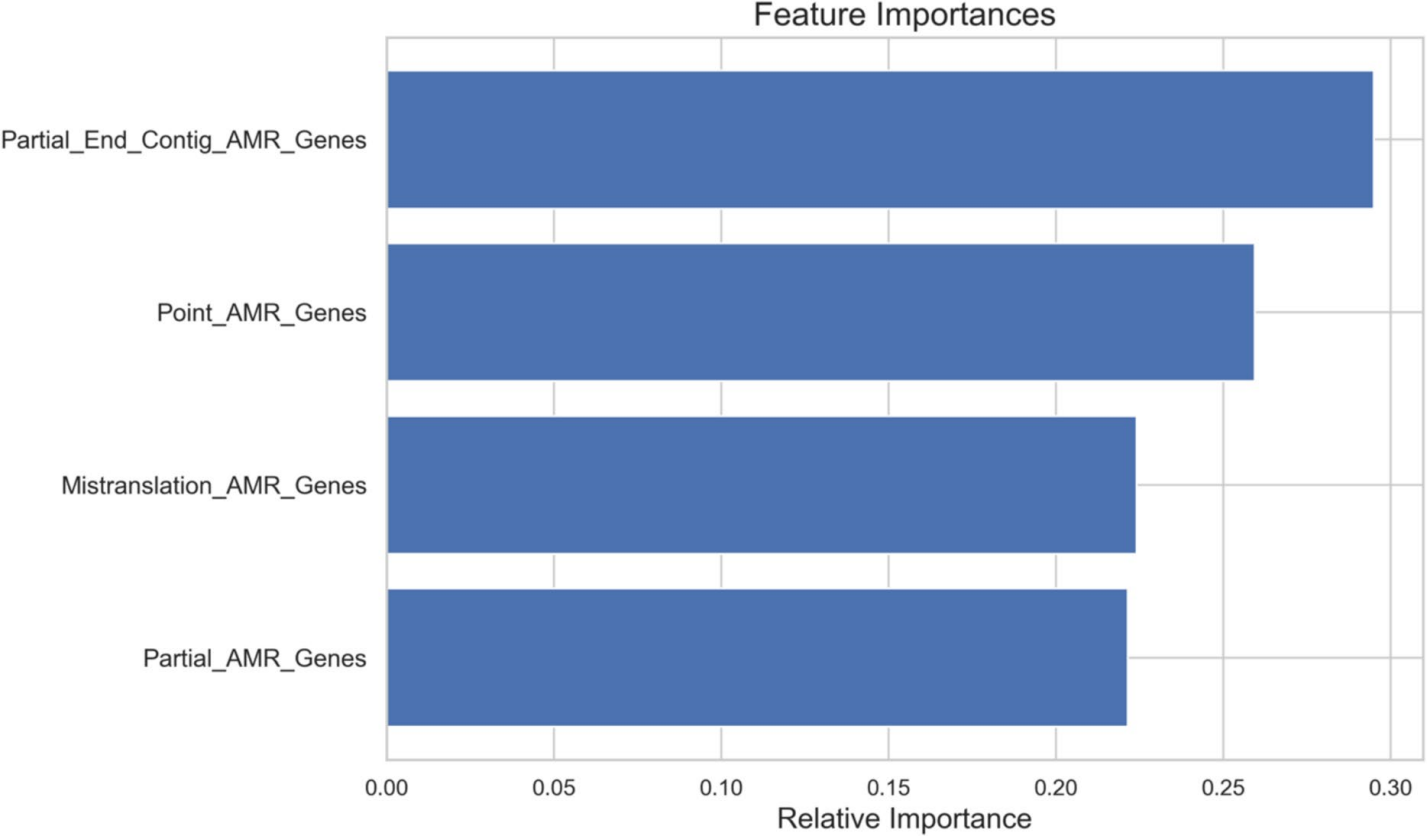
Feature importance in predicting complete AMR genes using Random Forest regression

The Random Forest model (Fig. 5) complements the regression analysis by handling non-linear relationships, offering additional validation of the findings. With an R-squared value of 0.936, the model demonstrates strong predictive capability, emphasising the significance of PECAGs (importance = 0.29) and PAGs (importance = 0.26) as the most critical predictors. These results connect directly to the study’s goal of identifying actionable predictors for AMR mitigation strategies.

## Discussion

The findings of this study provide compelling evidence that industrial activities significantly contribute to climate change, which, in turn, influences the distribution and frequency of antimicrobial resistance genes across various environments. These results align with and extend existing literature, highlighting the multifaceted relationship between industrialisation, climate change, and AMR.

The study found a strong correlation between industrialisation and global warming, with the top 20 highly industrialised nations experiencing temperature increases of up to 2.0 °C in 2023, while poorly industrialised nations exhibited a mean temperature increase ranging from 1.2 to 1.8 °C in 2023. However, the observation that many poorly industrialised nations exhibit temperature increments like those in highly industrialised regions suggests that global warming is driven by more than just local industrial emissions. Instead, the accumulation of greenhouse gases in the worldwide atmosphere creates a baseline warming effect that—when coupled with regional climatic factors such as atmospheric circulation patterns, ocean currents, and feedback mechanisms like ice-albedo and water vapor feedback exacerbates local temperature increases (Anderson et al., 2016; Cai et al., 2021)—results in comparable temperature increases even in regions with lower levels of industrial activity. Prior research supports this multifaceted relationship, highlighting that while industrialisation is a primary contributor to greenhouse gas emissions (Denchak, 2023; U.S. Environmental Protection Agency, 2024), global climate dynamics such as ocean circulation, atmospheric circulation patterns, and solar radiation variability also play a critical role in determining regional temperature increments (Intergovernmental Panel On Climate Change (IPCC), 2023). For instance, regions like Europe, which hosts numerous industrialised nations, have experienced disproportionate temperature rises exceeding + 2.0 °C in some areas by 2020, further corroborating the complex interplay between industrial activity and climate change.

Climate change has profound implications for bacterial physiology and the evolution of resistance. Rising temperatures and altered ecosystems increase the survival and adaptability of bacteria, potentially mimicking the effects of antibiotic exposure (Burnham, 2021; MacFadden et al., 2018). This environmental pressure accelerates the proliferation of AMR genes, suggesting that climate change impacts ecological balance and directly influences the evolution and spread of resistance.

The disparity in AMR gene prevalence between highly and poorly industrialised nations reflects not only the differential impacts of industrialisation and resource availability but also differences in sequencing efforts and research output. Highly industrialised countries like the USA report AMR gene frequencies exceeding 130,000 complete AMR genes, likely due to extensive antibiotic use in healthcare and agriculture, as well as advanced surveillance and research infrastructure. This aligns with previous studies showing that industrialised nations have higher rates of antibiotic consumption and environmental pollution, contributing to the amplification of AMR (Barathe et al., 2024; Klein et al., 2018).

In contrast, underdeveloped African nations like Somalia and Burundi report significantly lower AMR gene frequencies. However, these findings may not indicate a true lower prevalence but rather highlight systemic limitations, including inadequate healthcare infrastructure, poor antibiotic surveillance, and restricted funding for AMR research (Gulumbe et al., 2022). The lack of robust data collection and monitoring systems calls attention to the urgent need for international support to address these challenges in resource-limited settings.

*E. coli* was chosen as the model organism for its clinical relevance and ubiquity in human and environmental microbiomes. As a significant cause of bacterial infections worldwide, *E. coli* is an ideal proxy for studying the dynamics of AMR gene distribution across regions with varying levels of industrialisation. Its well-characterised genome and widespread presence in industrial, agricultural, and clinical settings provide valuable insights into the broader impacts of industrialisation and climate change on microbial resistance.

The concept of ‘Eco-AMR Zones’ emerges as a promising strategy to tackle the interconnected challenges of AMR and environmental sustainability. These zones would target regions characterised by high levels of industrial activity and substantial antibiotic use, implementing strict pollution controls, sustainable manufacturing practices, and regulated antibiotic use in healthcare and agriculture. To facilitate practical implementation, regulatory frameworks could be developed to enforce emission standards, promote green technologies, and establish monitoring systems for antibiotic use. Examples of existing successful initiatives, such as the EU’s circular economy framework and Japan’s advanced wastewater treatment regulations (Hartley et al., 2023; Japan International Cooperation Agency, 2017), could serve as models for creating these zones. Such initiatives are consistent with prior recommendations for integrated approaches to AMR mitigation and environmental management (Aslam et al., 2024; Manyi-Loh et al., 2018; Nijsingh et al., 2019; Pruden et al., 2013).

Eco-AMR Zones could leverage cleaner technologies and promote public awareness to reduce the environmental footprint of industrial activities. By addressing environmental pollution, these zones could also directly mitigate public health risks associated with AMR, such as the spread of resistant infections and the increased burden on healthcare systems. Additionally, these zones could enhance global surveillance of AMR by establishing robust data collection and monitoring systems, potentially through international collaborations or by leveraging emerging technologies such as real-time environmental monitoring. However, the implementation of these zones may face challenges such as resistance from industries due to the cost of regulation, logistical difficulties in enforcement, and the need for robust infrastructure, particularly in low-resource settings. Collaboration among policymakers, healthcare professionals, environmental scientists, and industry leaders would be essential to overcome these hurdles and ensure their success. While establishing these zones requires substantial investment and may encounter initial resistance, the long-term benefits—improved public health through reduced AMR-related infections, reduced environmental degradation, and prolonged antibiotic effectiveness—justify the effort.

One unexpected finding was the relatively weak influence of certain AMR gene types, such as PECAGs, on the prevalence of CAGs. This may indicate that localised factors influence these resistance mechanisms more than overarching industrialisation trends. The variability in AMR gene diversity among industrialised nations also warrants further investigation. For instance, while the USA and the UK report disproportionately high AMR gene frequencies, countries like Germany and South Korea exhibit much lower counts. These differences may be attributed to variations in antibiotic stewardship policies, environmental regulations, and healthcare practices. The strong correlation between industrialisation and AMR gene prevalence underscores the need for comprehensive public health strategies incorporating environmental considerations. Policies to reduce industrial emissions, regulate antibiotic use, and enhance global surveillance systems are critical to mitigating the dual threats of AMR and climate change.

International collaboration will be essential to address these interconnected challenges. Industrialised nations must lead by adopting sustainable practices and supporting resource-limited regions in building AMR monitoring and intervention capacity. Incorporating evidence-based environmental policies into global AMR strategies is a public health imperative and a moral responsibility in safeguarding ecological integrity for future generations.

## Conclusion

This study highlights the interconnected challenges of AMR, climate change, and industrialisation, revealing critical disparities in their global impacts. Highly industrialised nations contribute significantly to global warming, accelerating the evolution and distribution of AMR genes. These nations exhibit higher AMR gene frequencies due to extensive antibiotic use, advanced healthcare systems, and industrial waste. In contrast, poorly industrialised nations face limited surveillance and resource constraints, making their actual AMR burden challenging to assess.

The study introduces the concept of Eco-AMR Zones, targeted areas focused on mitigating AMR and promoting sustainable practices in high-risk regions. These zones could address the dual threats of AMR and climate change by implementing pollution controls, regulating antibiotic use, and fostering stakeholder collaboration. Policymakers and industries are urged to adopt advanced wastewater treatment, enforce antibiotic monitoring systems, and incentivise non-antibiotic alternatives.

Global collaboration is essential, with developed nations providing financial and technical support to underdeveloped regions to strengthen AMR surveillance and infrastructure. The study also calls for future research to explore climate change’s role in AMR evolution, assess Eco-AMR Zones, and develop economic incentives for sustainable practices. By integrating public health and environmental strategies, stakeholders can address these intertwined global challenges. Immediate action and sustained investment are essential to protect human health, ensure ecological balance, and secure a sustainable future in the face of AMR and climate change.

## Data Availability

The dataset supporting the conclusions of this article is available on request.
